# Secretion of small proteins is species‐specific within *Aspergillus* sp

**DOI:** 10.1111/1751-7915.12361

**Published:** 2016-05-07

**Authors:** Nicolas Valette, Isabelle Benoit‐Gelber, Marcos Di Falco, Ad Wiebenga, Ronald P. de Vries, Eric Gelhaye, Mélanie Morel‐Rouhier

**Affiliations:** ^1^ Faculté des Sciences et Technologies BP 70239UMR1136 INRA‐Université de Lorraine “Interactions Arbres/Micro‐organismes”Université de LorraineVandoeuvre‐lès‐Nancy CedexF‐54506France; ^2^ Faculté des Sciences et Technologies BP 70239UMR1136 INRA‐Université de Lorraine “Interactions Arbres/Micro‐organismes”INRAVandoeuvre‐lès‐Nancy CedexF‐54506France; ^3^ Fungal PhysiologyCBS‐KNAW Fungal Biodiversity Centre & Fungal Molecular PhysiologyUtrecht UniversityUppsalalaan 8Utrecht3584 CTThe Netherlands; ^4^ Center for Structural and Functional GenomicsConcordia University7141 Sherbrooke Street WestMontrealQCH4B 1R6Canada

## Abstract

Small secreted proteins (SSP) have been defined as proteins containing a signal peptide and a sequence of less than 300 amino acids. In this analysis, we have compared the secretion pattern of SSPs among eight aspergilli species in the context of plant biomass degradation and have highlighted putative interesting candidates that could be involved in the degradative process or in the strategies developed by fungi to resist the associated stress that could be due to the toxicity of some aromatic compounds or reactive oxygen species released during degradation. Among these candidates, for example, some stress‐related superoxide dismutases or some hydrophobic surface binding proteins (HsbA) are specifically secreted according to the species . Since these latter proteins are able to recruit lytic enzymes to the surface of hydrophobic solid materials and promote their degradation, a synergistic action of HsbA with the degradative system may be considered and need further investigations. These SSPs could have great applications in biotechnology by optimizing the efficiency of the enzymatic systems for biomass degradation.

## Introduction

During the last decades, the expansion of large‐scale analyses revealed that fungi, independently from their lifestyle, excrete small proteins, designed as small secreted proteins (SSP) containing a signal peptide and a sequence of less than 300 amino acids. Only few of them have been functionally characterized. For example, in *Laccaria bicolor*, an ectomycorrhizal fungus (ECM), Missp7 has a role in symbiosis establishment by suppressing the plant defence reactions (Plett *et al*., 2011). In pathogenic fungi, some of these proteins, referred to as ‘effectors’, are key factors of infection since they are able to suppress plant defence responses and modulate plant physiology to accommodate fungal invaders and provide them with nutrients (Dodds *et al*., 2009; Presti *et al*., 2015). In saprobic fungi, some of them could be involved in the degradative capabilities of fungi as described for the *Trichoderma reesei* swollenin, that depolymerizes cellulose, facilitating the further action of carbohydrate‐degrading enzymes (CAZymes) (Saloheimo *et al*., 2002). Moreover, due to the small size of some of them, particular CAZymes or other degradative enzymes could be part of SSPs. Another example showed that several genes coding for SSP are induced in the ligninolytic fungus *Phanerochaete chrysosporium* grown in the presence of oak extracts (Thuillier *et al*., 2014). Some SSPs are more ubiquitous, being found in ECM, pathogenic and saprobic fungi. This is, for example, the case for hydrophobins (Wösten, 2001). These small proteins have been first described as surface proteins that enhance growth of aerial hyphae by lowering surface tension between interface of air and water (Wösten *et al*., 1999). Then, additional roles have been highlighted. In particular, hydrophobins can stimulate enzymatic hydrolysis of poly(ethylene terephthalate) in *Trichoderma* spp. (Espino‐Rammer *et al*., 2013). Additionally to alter the physicochemical properties of surfaces, they may also be able to physically bind degradative enzyme as cutinase and induce changes in the conformation of its active centre to increase activity (Ribitsch *et al*., 2015). In aspergilli, most SSP‐related studies have focused on the characterization of antimicrobial proteins because of their potential use in the combat against fungal contaminations and infections (Marx, 2004; Meyer, 2008). However, aspergilli have a great potential in plant biomass degradation through the production of lignocellulose‐degrading enzymes that are valuable for the bioenergy industry (Culleton *et al*., 2013; Liu *et al*., 2013; Miao *et al*., 2015). Yet, optimization of the efficiency of these enzymatic systems is required for industrial applications. We suggest here that SSPs, which have not yet been functionally characterized, could be a reservoir of new functions of interest related to the degradative properties of fungi.

In this study, we have performed a comparative analysis of both the copy numbers of SSP‐coding genes and the occurrence of the corresponding proteins within secretome data obtained previously for saprobic fungi with biotechnological interest: *Aspergillus fischeri*,* Aspergillus niger*,* Aspergillus nidulans*,* Aspergillus clavatus*,* Aspergillus fumigatus*,* Aspergillus terreus*,* Aspergillus oryzae* and *Aspergillus flavus* (Benoit *et al*., 2015).

## Material and methods

### Identification of SSP genes in Aspergilli genomes

Prediction of SSP‐coding genes was performed in genomes of eight aspergilli: *A. niger* ATCC1015, *A. fumigatus* af293, *A. clavatus* NRRL 1, *A. flavus* NRRL 3357, *A. oryzae* RIB40, *A. nidulans* AspGD, *A. terreus* NIH 2624 and *A. fischeri* NRRL181, available at the Joint Genome Database (JGI) (http://genome.jgi.doe.gov/programs/fungi/index.jsf) (Grigoriev *et al*., 2011). The prediction was performed using a custom bioinformatic pipeline described previously (Pellegrin *et al*., 2015). Briefly, genes were considered as SSP‐coding genes if (i) a signal peptide was detected in the sequence using SignalP with D‐cutoff values set to ‘sensitive’ (version 4.1; option eukaryotic; Petersen *et al*., 2011) and if (ii) the sequence was smaller than 300 amino acids.

### Comparative analysis of SSP in Aspergilli secretomes

To ascertain the occurrence of SSPs within the secretomes of the various aspergilli, we queried mass spectrometry proteomics data (available at ProteomeXchange Consortium, http://proteomecentral.proteomeexchange.org, with the dataset identifier PXD000982) against the predicted SSP set using blastp. This proteomic analysis has been performed for the eight aspergilli grown on either sugar beet pulp (SBP) or wheat bran (WB) (Benoit *et al*., 2015). The quantity of SSPs for both substrates was reported in Table S1 as the area under curve normalized with bovine serum albumin (BSA) signal, for two independent experiments. The orthologues of each SSP has been searched within the other aspergilli genomes using blastp, and accession numbers corresponding to the best hit proteins, such as their occurrence in the secretomes have been reported in Table S1. These data are presented in a principal component analysis (PCA) using a matrix based on the BSA normalized area under curve values as input. PCA was performed using xlstat and graphical representation using statistica.

### Phylogenetic analysis

Sequences of hydrophobic surface binding protein A (HsbA) were obtained from JGI database (http://www.ncbi.nlm.nih.gov/) using blastp with the sequences retrieved from the proteomic analysis, and *A. oryzae* (Prot ID 4766) and *A. niger* (Prot ID 1180625) sequences as input (Ohtaki *et al*., 2006; Delmas *et al*., 2012). Amino acid sequence alignments were performed with Muscle in mega version 5 (Tamura *et al*., 2011). The evolutionary history was inferred using the neighbor‐joining method (Saitou and Nei, 1987). Bootstrap tests were conducted using 500 replicates.

### Water activity measurement

The water activity (a_w_) of the two non‐inoculated media, SBP and WB, were measured after autoclave using a Novasina labmaster‐a_w_ (Novasina, Lachen, Switzerland).

### Sugar quantification

Individual sugar concentrations were determined by HPLC (Thermo Scientific 5000+ HPLC‐PAD system; Thermo Fisher Scientific Inc Waltham, Massachusetts, USA) using a multistep gradient. A flow rate of 0.3 ml min^−1^ was used on a CarboPac PA1 column (Guard column: Dionex CarboPac PA1 BioLC 2 × 50 mm and main column: Dionex CarboPac PA1 BioLC 2 × 250 mm). The column was equilibrated before injection with a pre flow of 18 mM sodium hydroxide (NaOH). During a total running time of 50 min, the following solutions were used: A, water; B, 100 mM NaOH; C, 100 mM NaOH with 1 M sodium acetate. During the first 20 min, 18% of B was applied, followed by a 10 min linear gradient to 40% C and 0% B, and 100% C for 5 min. To rinse the acetate, 100% B was used for 5 min, and 10 min of 18% B was used to rinse the column. The quantification was performed based on external standard calibration. Reference sugars (Sigma‐Aldrich, Zwijndrecht, Netherlands) were used in a concentration range from 2.5 to 200 μM. The data obtained are the results of two independent biological replicates and for each replicate three technical replicates were assayed.

## Results and discussion

### SSP‐gene copy numbers in Aspergilli

In aspergilli, according to the species, SSP‐coding genes represent between 2% and 3% of the predicted gene models (Table 1). This similar percentage among the eight aspergilli studied here, suggests a correlation between genome size and copy numbers of SSP‐coding genes. These percentages are similar to those calculated for the ligninolytic fungi *P. chrysosporium* (2.6%) and *Trametes versicolor* (2.4%) or the ectomycorrhizal fungus *L. bicolor* (2.1%) (Pellegrin *et al*., 2015). However, when SSP copy numbers are considered independently of the number of predicted gene models, huge differences were revealed among species (from 205 for *A. fumigatus* to 398 for *A. flavus*).

**Table 1:** Genomic and proteomic analysis of SSP in aspergilli genomes and secretomes in comparison with *Phanerochaete chrysosporium*,* Trametes versicolor* and *Laccaria bicolor*

Species	Genome size (Mbp)	Gene models	Number of SSP‐coding genes^a^	Number of SSPs in the secretomes^b^	Proportion of SSPs within total secretomes^c^
*A. fumigatus*	29.39	9781	205 (2.0%)	41 (20.0%)	6.6%
*A. clavatus*	27.86	9121	236 (2.5%)	23 (9.7%)	6.2%
*A. nidulans*	30.48	10 680	248 (2.3%)	34 (13.7%)	6.0%
*P. chrysosporium*	35.15	13 602	257 (2.6%)		
*A. fischeri*	32.55	10 406	263 (2.5%)	21 (7.9%)	4.4%
*A. niger*	34.85	11 910	269 (2.2%)	28 (10.4%)	5.1%
*A. terreus*	29.33	10 406	289 (2.7%)	52 (18.0%)	8.6%
*A. oryzae*	37.88	12 030	337 (2.8%)	50 (14.8%)	11.3%
*T. versicolor*	44.79	14 296	340 (2.4%)		
*A. flavus*	36.79	12 604	398 (3.1%)	59 (14.8%)	12.0%
*L. bicolor*	60.71	23 132	486 (2.1%)		

a Percentages are calculated according to the total of SSP‐coding genes.

b Percentages are calculated according to the total of SSP‐coding genes.

c Percentages are calculated according to the total of proteins identified by mass spectrometry in Aspergilli secretomes.

### Proteomic analysis of SSP

**Figure 1:**
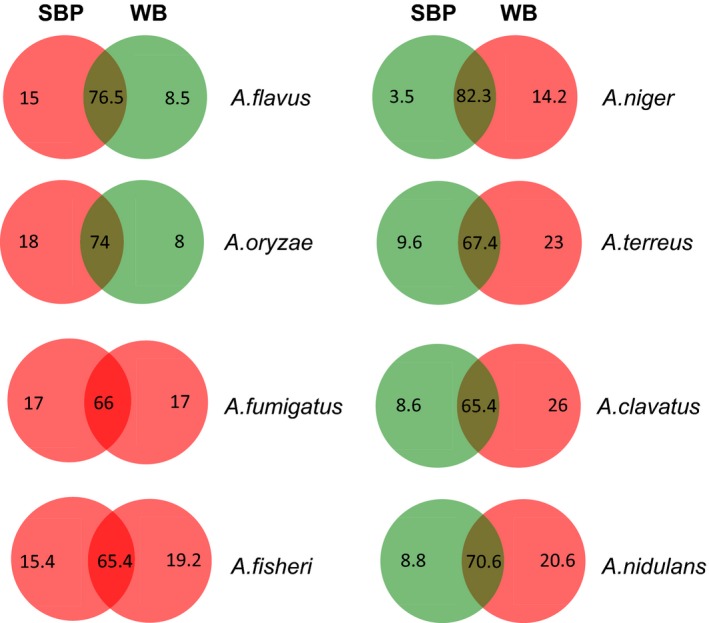
Venn diagrams showing the percentage of SSPs secreted on sugar beet pulp (SBP) or wheat bran (WB) or both. Red and green colours correspond, respectively, to percentages higher and lower than 10% of the total SSP for a species. Details concerning isoform annotation and peptide quantification are given in Table S2.

We propose here to analyse the occurrence of these SSPs in the secretomes of saprobic aspergilli in the context of biomass degradation. In a previous study, total proteins present in the secretomes of eight aspergilli were compared by mass spectrometry analysis during growth on either SBP or WB (Benoit *et al*., 2015). Between 8% and 20% of the predicted SSP‐coding genes were identified as proteins in the aspergilli secretomes (Table 1). Moreover, we showed that the proportion of SSPs among the total of secreted proteins, combining both conditions, varied for the species, *A. flavus* secreting the higher number of SSP. While most of SSPs were secreted in both conditions, some are specifically secreted depending on the medium (Fig. 1 and Table S2). Among them, some glycoside hydrolases, pectate lyases, esterases or lipases have been identified. Moreover, other proteins not directly involved in substrate degradation exhibit substrate specificities, such as allergens, hydrophobins and HsbA.

**Figure 2:**
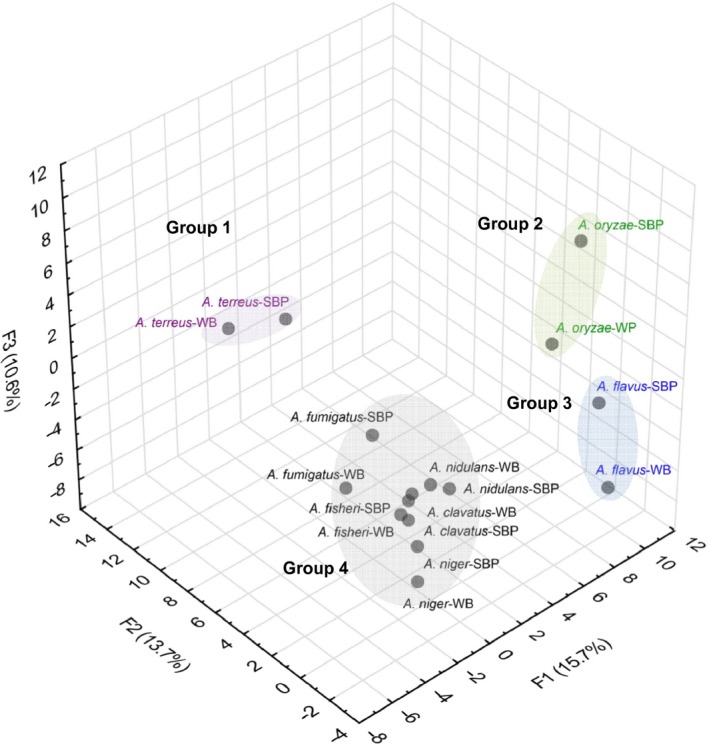
Principal component analysis plot showing the distribution of *Aspergillus* species based on their SSP secretion pattern. The values corresponding to the quantity of SSP secreted in sugar beet pulp (SBP) and wheat bran (WB) media were used as input (values reported in Table S1). The distribution of the proteins along F1, F2 and F3 axes explains the diversity for 15.7%, 13.7% and 10.6% respectively. These proteins are listed in Table S3. Four groups can be distinguished based on ANOVA analysis (data not shown).

To have a global view, a PCA was implemented to compare the species based on their SSP patterns. The quantity of secreted proteins (BSA normalized data of area under curve reported in Table S1) was used as input for the eight aspergilli. The proteins were found widespread in the graphical representation (F1, F2 and F3 accounted of 40% of the total variance) with a clear clustering into four groups, based on ANOVA analysis (Fig. 2). The distinct separation between the groups showed that the substrate is not the factor explaining the distribution; rather species secrete their own set of SSPs. Group 1 corresponds to *A. terreus*, group 2 corresponds to *A. oryzae*, group 3 corresponds to *A. flavus* and group 4 corresponds to *A. nidulans*,* A. niger*,* A. fischeri*,* A. clavatus* and *A. fumigatus*, suggesting specificities within secreted SSPs, especially for *A. terreus*,* A. oryzae* and *A. flavus* when compared with the others. In particular, the diversity between *A. terreus* and *A. flavus* is explained by the repartition of the proteins along the F1 and F2 axis, respectively, and the diversity between *A. oryzae* and the others is rather explained by the F3 axis.

### Hydrophobic surface binding proteins, HsbA

**Figure 3:**
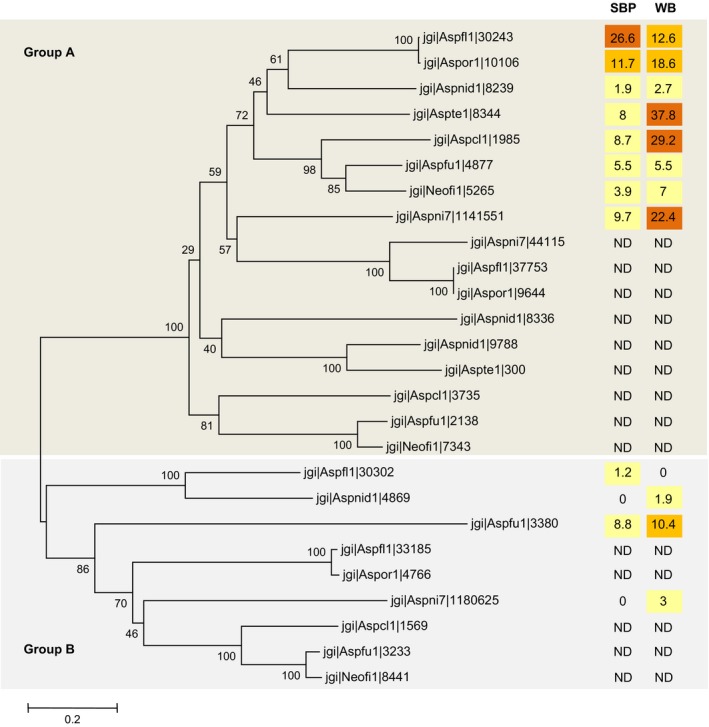
Neighbor joining phylogenetic tree of the HsbA coding genes identified in the genomes of the various aspergilli. The accession numbers are those retrieved from the JGI. The tree was constructed with MEGA5 (Tamura *et al*., 2011). Aspfl1: *A. flavus*, Aspor1: *A. oryzae*, Aspnid1: *A. nidulans*, Aspte1: *A. terreus*, Aspcl1: *A. clavatus*, Aspfu1: *A. fumigatus*, Neofi1: *A. fisheri*, Aspni7: *A. niger*. Bootstrap values are reported and the scale marker represents 0.2 substitutions per residue. Quantity of secreted proteins are reported as normalized area under curve for both substrates (SBP: sugar beet pulp and WB: Wheat bran) (See Table S1). The values correspond to the mean of two experiments.

The contribution of the various SSPs to this repartition is given in Table S3. Among the best candidates that could explain the diversity, some CAZymes, cutinase, HsbA, superoxide dismutase and hypothetical proteins can be pointed out. Moreover, the secretion of some of these proteins is species specific (Table S4). Interestingly, *A. terreus*,* A. flavus* and *A. niger* specifically secrete HsbA. HsbA is a small protein able to recruit lytic enzymes to the surface of hydrophobic solid materials and promote their degradation (Ohtaki *et al*., 2006). As an example, HsbA of *A. oryzae* has been shown to associate with the synthetic polyester polybutylene succinatecoadipate and promote its degradation through the recruitment of a specific polyesterase (CutL1). In *A. niger*, two genes encoding hydrophobin family proteins and one HsbA were strongly induced by the switch from glucose to wheat straw suggesting that these proteins could have also a role in recruiting degradative enzymes to the straw surface (Delmas *et al*., 2012). From two to four genes with HsbA domains have been identified within the genomes of aspergilli. The phylogenetic analysis of the amino acid sequences revealed two distinct clusters that we have identified as group A and B in Figure 3. The sequences coding for the isoforms that have been functionally characterized in *A. oryzae* (Ohtaki *et al*., 2006) and in *A. niger* (Delmas *et al*., 2012) belong to group B, however, they are not (or very few) secreted by the tested fungi on SBP and WB. By contrast, one isoform for each species belonging to group A is secreted in both conditions at variable amounts. The isoform of *A. flavus* (prot ID 30243) is preferentially secreted on SBP, while the ones from *A. terreus* (Prot ID 8344), *A. clavatus* (Prot ID 1985) and *A. niger* (Prot ID 1141551) are produced on WB. Considering both the specificities of aspergilli regarding their biomass degradative systems previously highlighted (Benoit *et al*., 2015), and the described role of HsbA as helper‐proteins for degradation, a synergistic action of these proteins may be considered and needs further investigation.

### SSP as stress‐related proteins

*Aspergillus* species grow well in sugar‐rich habitats and are thus highly tolerant in relation to solute‐induced stresses (Chin *et al*., 2010; de Lima Alves *et al*., 2015). Although SBP and WB are mostly made of polysaccharides, the sugar analysis of these two substrates after autoclaving and before inoculation with the fungi (see material and methods), revealed a relatively poor free‐sugar content. Only glucose, fructose and sucrose were detected. Glucose and fructose concentrations were very low and similar between both substrates (Table 2). The concentration of sucrose was significant and twice as high in SBP as in WB, but far from the molar concentration that can be found in sugar‐rich habitats (Lievens *et al*., 2015). In accordance, measuring the water activity (ɑ_w_) revealed no significant difference between the two media, which both exhibit a very high water activity. However, aspergilli are mostly xerophilic and optimal growth could be observed for low ɑ_w_, as for two strains of *A. penicilliodes*, which are capable of mycelial growth down to a water activity of 0.647 ɑ_w_, with an optimal growth between 0.800 and 0.820 ɑ_w_ (Stevenson *et al*., 2015). According to these results, it could be pointed out that the culture conditions used in this study could be stressful for fungi when compared with their natural habitat. Accordingly, extracellular superoxide dismutase has been highlighted within the aspergilli secretomes, especially for *A. terreus*,* A. flavus*,* A. oryzae* and *A. nidulans* (Table S1). The secretion of some SSPs could thus be a way to resist stress as suggested for the lignolytic fungus *P. chrysosporium* that induces expression of several SSP‐coding genes in the presence of toxic oak extracts (Thuillier *et al*., 2014).

**Table 2:** Free sugar composition and water activity of the wheat bran and sugar beet pulp media. Sugar concentrations are given in millimolar. Details are given in Materials and methods part

Substrate	Glucose	Fructose	Sucrose	a_w_
Wheat bran	0.3	0.2	10.5	0.990 ± 0.001
Sugar beet pulp	0.5	0.2	20.2	0.992 ± 0.002

## Conclusion

In this study, we show that, similar to the degradative enzymatic system, the secretion of non‐CAZy SSPs is fungal species dependent. Our hypothesis is that these proteins could participate to plant biomass degradation, with a similar process as described for the hydrophobins or HsbA. SSPs could recruit enzymes at the surface of the substrate or directly interact with the enzymes to increase their activity. They may also be involved in the strategy developed by fungi to resist the stress that could be due to the toxicity of some aromatic compounds or reactive oxygen species released during the degradative process. The SSP candidates highlighted in this study are mostly functionally uncharacterized and are therefore an interesting potential source of new functions for plant biomass conversion.

## Conflict of Interest

The authors declare no conflict of interest.
